# The United States and the world economy in the future: America’s global exceptionalism firmly rooted in entrepreneurship

**DOI:** 10.4103/2152-7806.66459

**Published:** 2010-07-16

**Authors:** Morris Beschloss

**Affiliations:** Rancho Mirage, USA

Morris Beschloss is a former corporate CEO, an economic analyst, writer and lecturer. He appears on television and radio and has a daily blog in newspapers across the USA. I asked him to write about the future of the economies of the countries around the world in the next 5 to 10 years. This is his response.

As a background for our readers, Keynesian economic theory was developed by John Manyard Keynes, a British economist in the 1930s. Keynes believed that increased government spending during economic recessions could revive the economy. According to Dick Armey, a Professor of Economics and former Member of the US Congress (WSJ; February 4, 2009; “Washington could use less Keynes and more Hayek”), Keynes believed that “general employment was always positively correlated with the aggregate demand for consumer goods. Keynes argued that government should intervene in the economy to maintain aggregate demand and full employment, with the goal of smoothing out business cycles. During recessions, he asserted, government should borrow money and spend it.” His macroeconomic theories have been adopted by most western countries and have been embraced by the Obama administration in the USA. His policies are also the basis of Chinese economics, which is Marxist in origin.

Friedrich Hayek, a contemporary of Keynes and a Noble economist from Austria, disagreed with Keynes and supported classic economic theory that advocated “balanced budgets and government restraint.” Another Nobel economist, James Buchanan, argued “that the great flaw in Keynesian economics is that it ignores the obvious self-interested incentives of government actors implementing fiscal policy and creates intellectual cover for what would otherwise be viewed as self-serving and irresponsible behavior by politicians. It is also very difficult to turn off the spigot in better economic times, and Keynes blithely ignored the long-term effects of financing an expanded deficit.” Armey stated that “if the government borrows the money for the stimulus, then it will either have to print money later or raise taxes to pay it back. If the government prints the money, it will increase inflation, which will decrease the value of the dollar … Hayek viewed the boom and bust of the business cycle as primarily a monetary phenomenon created by government’s artificial inflation of money and credit … The problem with government attempts to manipulate the economy through fiscal policy — spending that takes resources away from those who are productive and redistributes it to politically-favored interests — is that they are audacious. It assumes that government knows better how to spend and invest than individuals acting in their families’ best interest … no one spends someone else’s money better than they spend their own.”

So, those are the opposing macroeconomic theories that are influencing the economies of the world today. What is happening in your country now and how will those changes affect your practice of medicine? Read Morrie’s comments below.

**— Editor-in-Chief**

## AMERICA’S GLOBAL EXCEPTIONALISM FIRMLY ROOTED IN ENTREPRENEURSHIP

The most remarkable evidence of America’s global economic leadership is the preponderance of private businesses that comprise the backbone of the nation’s success, since its inception in the late 18^th^ century.

Although current statistics don’t tell the whole story, they provide a focus on what has made the U.S.A. the world leader in all aspects of economic progress, whether total annual generation of gross domestic product of goods and services, consumer demand, per capita income, and even world trade. This relatively recent venture into exports has vaulted the U.S. into the runner-up spot among China, Germany and Japan, whose economies are predominantly dependent on outgoing shipments. While those countries’ exports comprise a major percentage of their productive capacity, America’s export sector comprises only 12% of the nation’s total revenues.

The following key statistics emphasize America’s overwhelming proportion of the world’s gross domestic product of goods and services:

1) With a population of less than five percent of the world’s total, the U.S. generates almost 24% of global gross domestic product.
2) The U.S. financial markets, both liquid and invested world assets, comprise 25% of all of the world’s financial holdings and transactions.
3) America is considered the world’s ultimate safe haven, especially at times of international stress, which is currently underway. America has never reneged on repayment of debt or interest on the balance. This factor has driven the value of the dollar versus a basket of the world’s leading currencies to the highest value in the past two years.
4) America is the acknowledged world leader in technological and innovative evolution, and still ranks ahead of China and other nations in total manufacturing revenues. This is true despite the loss through worldwide displacement of such major industries as automotive, steel, electronics, metal fabrication, textiles, etc.
  However, paramount in the hopeful continuation of America’s economic world mastery is the entrepreneurship of the nation’s talented individualism.
5) Despite the increasing antipathy toward the use of this nation’s abundant natural resource extraction, such as coal, oil and natural gas, America is in the forefront of renewable energy development such as solar, geothermal and wind power.
6) Sixty-five percent of all gainfully employed Americans work for themselves or within privately-owned companies. While the once dominant western European nations and Japan held the key to the past century’s unprecedented industrial, commercial, and innovative progress, their increasing commitment toward government control and progress involvement have created untenable obstacles to the individualistic creative capability that has marked the forward drive of these nations’ once unchallenged supremacy.

Utilizing the Keynesian concept of leveling the playing field, and removing the benefits of superior entrepreneurship through increasingly oppressive tax policies, the western nations, plus Japan, have throttled the human tendency to attain rewards in lock-step with individual achievement. This motivational philosophy has been replaced with affirmative action and similar leveling strategies that are increasingly Marxist in their implementation.

They suspiciously echo the credo of Karl Marx, who uttered the defining axiom of his newfound method of egalitarianism memorializing “those according to their means to those according to their needs.”

This approach has already squeezed much of the world’s creative juices and their just accomplishments dry, while putting viable nations behind debt-ridden eight balls. Although this is already decimating such once balanced-budget nations into an ever-deeper financial hole, it has methodically pushed the lower ranks of the Eurozone — Portugal, Ireland, Greece, Spain, and Italy — dangerously close to a point of no return.

Up to now, America has avoided the worst aspects of its transfiguration from entrepreneurial exceptionalism to the wealth redistribution concept, which has proven disastrous. This failed philosophy was behind the breakup of the Soviet Union, and more recently, the current fiscal nightmares occurring in the West. Present American government policies are leading the U.S.A. toward similar paths. But, fortunately, this country has only taken the initial steps, which can still be reversed by political changes, rejecting the utopian Marxist concepts and embracing the tried and true values that have made the 310 million–strong American nation the envy of the world at large.

Barring such return to fiscal sanity and rewarding the millions of American achievers through the reinstallation of economic systems that reached their peak in the 1980s, it’s a sure bet that resource-rich and capitalist-minded developing nations will fill the leadership vacuum left by the world’s last standing, merit-rewarding nation, the U.S.

If such a departure indeed occurs, it will leave the dominance of world economics wide open. Along with such a development will come leadership in geopolitics, military potential, and a global system of human values far different than the one U.S. leadership has brought to the world at large in the past 65 years.

